# No alteration of back muscle oxygenation during isometric exercise in individuals with non-specific low back pain

**DOI:** 10.1038/s41598-022-11683-x

**Published:** 2022-05-18

**Authors:** Anke Langenfeld, Brigitte Wirth, Andrea Scherer-Vrana, Fabienne Riner, Kyra Gaehwiler, Paola Valdivieso, B. Kim Humphreys, Felix Scholkmann, Martin Flueck, Petra Schweinhardt

**Affiliations:** 1 Department of Chiropractic Medicine, Balgrist University Hospital and University of Zurich, Forchstrasse 340, 8008 Zurich, Switzerland; 2 Scholkmann Data Analysis Services, Scientific Consulting and Physical Engineering, Schuppisstr. 5, 8057 Zurich, Switzerland; 3 Department for Neonatology, University Hospital Zurich and University of Zurich, Rämistrasse 100, 8091 Zurich, Switzerland; 4 Laboratory of Muscle Plasticity, Balgrist University Hospital and University of Zurich, Balgrist Campus, Lengghalde 5, 8008 Zurich, Switzerland; 5 Winterthur Institute of Health Economics, School of Management and Law, University of Applied Sciences, Gertrudstr. 15, 8400 Winterthur, Switzerland; 6 Present Address: Swiss Federal Institute of Sport Magglingen SFISM, Lärchenplatz building HLP 107, 2532 Magglingen, Switzerland

**Keywords:** Diseases, Medical research

## Abstract

The aim of our study was (I) To compare back muscle oxygenation and perfusion as well as Biering–Sorensen muscle endurance (BSME) test holding times between chronic non-specific low back pain (CNSLBP) patients and asymptomatic controls matched for age, body mass index (BMI), sex and physical activity, and (II) to investigate factors associated with BSME holding times. Muscle perfusion (tHb) and oxygenation (SmO_2_) were measured by near-infrared spectroscopy (NIRS) based oximetry in three back muscles during the BSME. Reliability of tHb and SmO_2_ was assessed in a separate sample. BSME holding time and SmO_2_ were compared between patients (n = 45) and controls (n = 45) and factors associated with BSME holding time were assessed using multiple linear regression. Reliability for SmO_2_ was excellent (ICC = 0.87–0.99). THb showed poor to moderate reliability and was not further used. Groups differed for BSME holding time (*P* = 0.03), pain intensity (*P* ≤ 0.0005) and subcutaneous tissue thickness (*P* = 0.01) but not for NIRS measures. Physical activity and BMI were associated with BSME holding times. Insufficient muscle oxygenation does not seem to be a major factor contributing to CNSLBP. Future investigation should evaluate other determinants of BSME holding times, such as motivation and recruitment of auxiliary muscles.

## Introduction

Low back pain (LBP) is a common condition, globally the number one cause for years lived with disability^1^. In most instances, no specific cause can be identified^1,2^, resulting in the unsatisfactory diagnostic category ‘non-specific LBP’ (NSLBP). Despite the absence of a clear cause, it has been reported that patients with NSLBP have altered muscle perfusion, resulting in decreased endurance capacity of paraspinal muscles^3,4^ and impaired muscle oxygenation^5,6^. In fact, it has been observed that changes in oxygen levels in the erector spinae muscle during back flexion and extension were only altered in patients with NSLBP but not in patients with specific causes for LBP, such as structural abnormalities^4^. This might indicate that altered muscle perfusion and/or oxygen levels are patho-physiologically relevant in NSLBP. In rodents, it has been shown that already a slight increase in muscle metabolites (i.e. protons, lactate and adenosintriphosphate), as would be expected with reduced perfusion, can activate nociceptors^7^. The synergistic effect of these three metabolites in eliciting muscle pain has been confirmed in healthy human subjects^8^.

However, the majority of studies that investigated muscle perfusion in patients with (chronic) NSLBP (CNSLBP) used dynamic muscle tests^3,5,6^, although the assessment of isometric endurance capacity has been suggested to be the most appropriate method to examine muscle fatiguability in CNSLBP^9^. Indeed, CNSLBP is associated with a decrease in isometric endurance capacity^10,11^. The gold standard to assess the isometric endurance capacity of back muscles is the Biering–Sorensen Muscular Endurance test (BSME)^10,12^. Two studies that used the BSME in conjunction with near infrared spectroscopy (NIRS) based oximetry to measure changes in muscle perfusion and muscle oxygenation provided mixed results: one study did not report differences in BSME holding times between CNSLBP patients and healthy controls, but found an increased recovery time of oxygenation in the patients^13^. The other study found significantly decreased holding times of CNSLBP patients in the BSME, but the muscle perfusion and oxygenation parameters did not differ between patients and controls (recovery time was not determined)^11^. The isometric endurance capacity as measured by the BSME is known to be influenced by age, sex, body mass index (BMI) and the subject's activity level^14–18^. However, the latter was not considered in these two studies. Therefore, the first aim of the present study was to compare BSME holding times as well as muscle oxygenation and perfusion during the BSME between CNSLBP patients and asymptomatic controls matched for physical activity, age, sex and BMI and controlling for subcutaneous tissue thickness.

Furthermore, changes in muscle oxygenation parameters during the BSME (the change from the minimum value during the BSME to the maximum value during recovery as well as the change during rest to the maximum or minimum value during the BSME) best predicted BSME holding times in a multiple regression model in healthy men (20). Similarly, the same muscle oxygenation parameters correlated with BSME holding times in physically active CNSLBP patients (13). In addition, it is conceivable that the cardiovascular response during the BSME^19^ influences BSME holding times. Therefore, the second aim of this study was to investigate the influence of muscle oxygenation on BSME holding times in addition to age, sex, physical activity, BMI, mean arterial pressure (MAP), pulse pressure (PP) and heart rate (HR) to better understand which factors determine the BSME holding times across CNSLBP patients and asymptomatic controls.

## Methods

This is an explorative observational study using an individually matched sample design. CNSLBP patients were compared to asymptomatic controls, individually matched for age, BMI, sex and physical activity as assessed by the Baecke Sportscore^20^. The study was approved by the ethics committee of the canton of Zurich, Switzerland (2016-00647) and registered at clinicaltrials.gov (NCT02955407). Each participant signed an informed consent prior to be included in the study. All experiments were performed in accordance with national and international guidelines and regulations, and the Declaration of Helsinki. Patients and asymptomatic controls were recruited via the Policlinic for Chiropractic Medicine at the University Hospital Balgrist, Zurich, Switzerland, through personal communication and digital distribution of study information among hospital staff as well as through advertisements at Zurich University, Zurich, Switzerland. Each subject signed an informed written consent before inclusion in the study.

### Participants

Eligibility criteria were age between 18 and 65 years and Caucasian origin (due to requirements of NIRS oximetry measurements). Caucasian origin was rated by the clinician conducting the entrance examination, asking about the European descendance of the test subjects’ parents. Exclusion criteria were prior spinal surgery, fractures, inflammation or tumor, any neurological pathologies, radiologically confirmed spinal instability, severe chronic diseases that impact physical activity, osteoporosis or cardiovascular diseases as well as pregnancy. Additionally, patients had to have LBP for more than three months, either continuously or recurrent episodes. Asymptomatic controls were excluded if they had LBP at the time of measurement or more than one episode of LBP lasting for more than one week previously in their life.

### Back muscle perfusion and oxygenation

**Figure 1:**
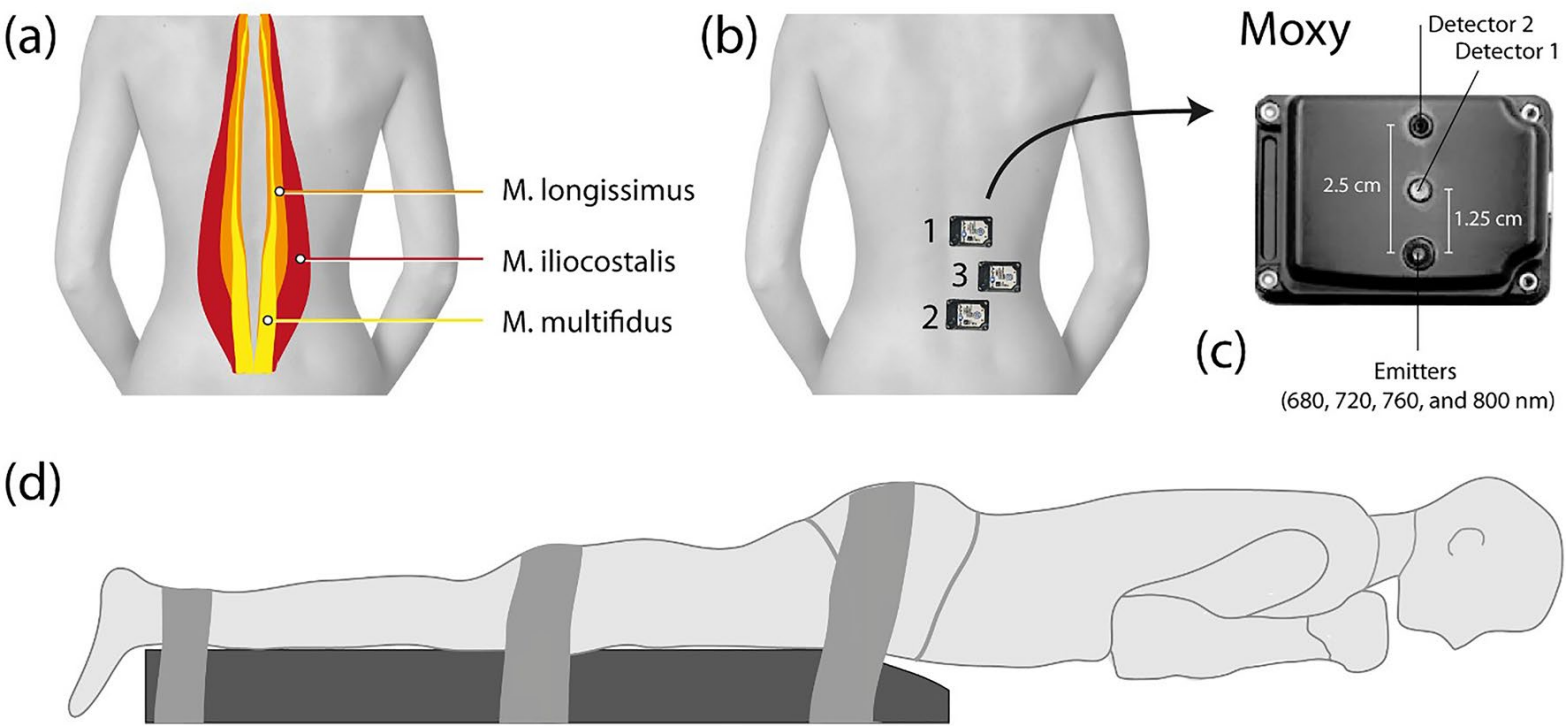
Positioning of the test subject and NIRS oximetry devices during the Biering–Sorensen muscle endurance (BSME) test. (**b**) Schematic representation of the location of *M. longissimus*, *M. iliocostalis* and *M. multifidus*, (**c**) positioning of the NIRS oximetry devices in the back, (**d**) close-up of the NIRS oximeter. (**d**) Visualization of a test subject performing the BSME test.

Blood perfusion of three back muscles (*M. longissimus*, *M. multifidus* and *M. iliocostalis*) was assessed using NIRS oximetry [Muscle Oxygen Monitor (Moxy) (Fortiori Design LLC, Minnesota, USA)] during the BSME. In a previous study the Moxy device was found to be more sensitive to changes in muscle oxygenation and perfusion compared to an alternative, therefore it was chosen as the measurement device in this study^21^. The probes were placed on the right side of the back as recommended by the SENIAM (Surface ElectroMyoGraphy for the NON-Invasive Assessment of Muscles) group (Fig. 1)^22^. For the *M. longissimus*, the probe was placed at two finger width laterally from the spinous process of the first lumbar vertebra (L1). For the *M. multifidus*, the electrode was placed on a line from the caudal tip of the posterior spina iliaca superior to the space between L1 and L2, at the level of the L5 spinous process (i.e. about 2–3 cm from the midline). For the iliocostalis muscle, the probe was placed one finger width medial from the line from the posterior spina iliaca superior to the lowest point of the lower rib, at the level of L2. The positions for the probes were palpated and marked in standing upright position. Because subcutaneous tissue is known to influence NIRS oximetry measurements^23^, its thickness was measured before the NIRS measurements. The experimenter held the subcutaneous tissue at the NIRS oximetry probe location for the *M. longissimus* and *M. multifidus* between two fingers and measured it using a caliper (GPM, Caliper, DKSH Switzerland Ltd, Zurich, Switzerland). Each location was measured three times and the average of the three measurements was used. Measurements above 13 mm resulted in exclusion of the subject from the study, as recommended in the manual provided by the manufacturer^24^.

### Biering–Sorensen muscle endurance test

For the BSME, the subject was lying prone on a treatment table with the pelvic bone on the edge of the table, the arms folded across the chest and the upper part of the body extending the treatment table. A 2D inclinometer (Noraxon U.S.A. INC, Arizona, USA) was placed at the level of the vertebra T7 in order to control the subject’s position during the test. The subject was instructed to hold the upper part of the body horizontally as long as possible. Deviations of more than five degrees from the horizontal testing position were signaled by a tone from the inclinometer. Required adjustments were instructed by the investigator. If the subject deviated from the horizontal position more than twice, the BSME test was stopped. After 240 s, the test was terminated by the investigator^25^. If subjects terminated early, the time and the reason for early cessation were noted.

### Rating of pain intensity

Participants were asked to rate their back pain intensity right before the measurement started on a numerical rating scale (NRS). Ranges from 0 to 10, 0 representing no pain at all and 10 the worst imaginable pain^26^. It is a valid and reliable tool to assess a patient`s perceived pain in various medical settings^26^.

### Baecke physical activity questionnaire

The Baecke physical activity questionnaire is a self-administered questionnaire about habitual physical activity^20^. It consists of sixteen items covering three components; work, sport and leisure-time. The items for work and leisure time are scored on a five-point Likert-scale ranging from 1 to 5. One indicating least activity and five indicating most activity. A total score can be calculated by summing up, work index, sport index and leisure index. The items for sport are divided into three sub-levels: time, intensity and proportion of the two most frequently played sports. A simple sport score can be calculated by the sum of intensity, time and proportion and is then converted to a five-point Likert-scale^20,27^. For subjects who do not perform any sport a rating of 0 is given^20^. The sport score of the questionnaire was used to assess subjects' physical activity levels and to match LBP subjects and asymptomatic controls.

### Systemic physiological measures

Blood pressure and heart rate were measured throughout the experiment non-invasively. The measurement device (SOMNOtouch NIBP SOMNOMedics GmbH, Randersacker, Germany) was attached at the fingertip and wrist. Based on these data the following parameters were calculated: mean arterial pressure (MAP), pulse pressure (PP) and heart rate (HR). MAP is defined as the average of arterial blood pressure during a cardiac cycle^28^. PP is defined as the difference between the systolic and diastolic blood pressures^29^.

### Experimental protocol

Before subjects were included in the study, they were examined by a chiropractor at the University Hospital Balgrist, Zurich, Switzerland, to check in- and exclusion criteria. Afterwards, the participant laid down on the treatment table prone, with the arms next to the body. Three straps were fixed around the pelvis, the knees and the ankles to avoid rotation of the legs (Fig. 1). The NIRS oximetry devices were attached on the corresponding marked position on the skin after wiping off the marker to avoid light absorption. The BSME was started after a baseline NIRS measurement of 2–3 min. After termination of the BSME, the subject stayed in prone position for another three minutes, during which the NIRS measurements were continued. To maintain a stable position during baseline and post-test measurements, the upper body of the participant was placed on two boxes which had the same height as the treatment table. After the baseline measurements, the treatment table was raised to lift the participants upper body off the boxes. At the end of the BSME test the treatment table was lowered to carry the upper body of the participant again.

### Reliability testing

To assess reliability of the NIRS measurements with this study’s set-up, 15 asymptomatic controls performed the BSME twice, following the same experimental protocol as described above, with a time interval of 7 days between the two measurements (maximal difference in time of day: 1 h).

### Data analysis

Muscle oxygenation was measured as the absolute tissue oxygen saturation (SmO_2_, measured in %); muscle perfusion was inferred from changes in total hemoglobin (tHb). SmO_2_ reflects the ratio between oxygen supply and consumption on the muscle level and is considered an accurate quantitation of oxygen changes in the muscle^30^, while tHb reflects the sum of the absolute concentrations of oxygenated hemoglobin (O_2_Hb) and deoxygenated hemoglobin (HHb) (tHb = O_2_Hb + HHb)^30^, which indicates changes in tissue blood volume and thus is influenced at short term only by perfusion. NIRS oximetry measurement data were processed by first interpolating missing values, resampling to 2 Hz and low-pass filtering by locally weighted regression fitting using a 2nd order polynomial (LOESS, window length (w) = 10 s). The following parameters were calculated for SmO_2_ and well as for tHb, based on changes relative to baseline, for SmO_2_ and well as tHb: area under the curve (AUC) for the whole trial (i.e. start until end of BSME), Median 1 (i.e. first minute after trial start to the second minute after trial start [duration 1 min]), Median 2 (i.e. time from the second minute after trial start until the end of the trial [variable duration across subjects]) and Median 3 (i.e. 10 s before the end of task to end of task [10 s]). The recovery time of SmO_2_ and tHb was defined as the time elapsed to reach 63.2% (corresponding to the exponential constant 1/e) of the post BSME maximum value. It was calculated by a non-linear regression analysis and a fitted exponential function. The calculated regression coefficient was used to define the time constant in seconds.

The NIRS data of the healthy controls who did the procedure twice were statistically analysed for test–retest reliability using the intraclass correlation coefficient (ICC)^31–33^ based on a 2-way mixed effects, single measurement, absolute agreement model^34^. Values of less than 0.5 were considered poor, between 0.5 and 0.75 moderate, and greater 0.75 excellent reliability^34^. Only measures with excellent reliability were kept for further analyses. Before subjecting the measures with excellent reliability to further analyses, they were corrected for subcutaneous tissue thickness, which differed significantly between groups and was significantly related to the NIRS measures. This was achieved by linear regression with NIRS measures as dependent variable and subcutaneous tissue thickness as independent variable and keeping the unstandardized residuals (termed in the following ‘corrected’). Clinical and demographic characteristics as well as BSME holding times and corrected NIRS measures of the CNSLBP patients and their matched controls were descriptively analyzed and compared using unpaired t-tests^35^. In addition, two-way ANCOVAs using corrected NIRS measures as dependent variables, gender and group as independent variables and BMI and age as covariates were performed to test whether there was an effect of group when gender, BMI and age were controlled for. Because a pain rating above 2 was used as an inclusion criteria in another study on BSME and NIRS in CNSLBP^13^, the group comparisons were repeated with a reduced sample of patients with pain intensity ratings above 2 at the day of examination and their matched asymptomatic controls. To assess the importance of possible influencing factors on BSME holding times as dependent variable, the following parameters were included as independent variables into a multiple regression model: muscle oxygenation (corrected SmO_2_), physical activity scores, BMI, pain intensity, MAP, PP and HR (continuous variables) as well as sex and group (LBP/asymptomatic controls) (categorical variables).

Data were collected and stored in the research electronic data capture (REDCap Version 9.6.1). The statistical analysis was performed using IBM SPSS Statistics (Version 26) statistical software package (SPSS, Inc., Chicago, IL, USA). The significance level was set for all analyses to α = 0.05.

## Results

### Reliability

15 asymptomatic controls (8 females, 7 males), mean age 35 years (SD 15 years), were included in the reliability study. Excellent reliability was found for SmO_2_ in the *M. longissimus* and *M. multifidus* (ICC = 0.87–0.99) for AUC, Median 1, Median 2 and Median 3 (Table 1). In contrast, poor reliability was found for SmO_2_ (AUC, Median 1, Median 2, Median 3) in the *M. iliocostalis* (ICC = 0.00–0.38). Data for *M. iliocostalis* were lacking in four subjects due to restricted space on the subjects' back or poor quality of the data. THb was found to have poor to moderate reliability in all examined muscles for AUC, Median 1, Median 2 and Median 3 (ICC = 0.11–0.72) and was therefore excluded from further analysis. The ICC for recovery time could only be calculated for SmO_2_ of the *M. longissimus* and *M. multifidus* because data for *M. iliocostalis* and tHb in general was often missing due to poor quality of the data. The reliability for these measures was poor (ICC = 0.25 and − 0.22).

**Table 1:** Test–retest reliability for area under the curve (AUC), Median 1, Median 2, Median 3 for oxygen saturation (SmO_2_) perfusion (tHb) and recovery time of *M. longissimus*, *M. multifidus* and *M. iliocostalis*.

	ICC	95% CI	
		LL	UL
**AUC_SmO**_**2**_**_M.longissimus**	**0.912**	**0.720**	**0.975**
**AUC_SmO**_**2**_**_M.multifidus**	**0.868**	**0.555**	**0.966**
AUC_SmO_2__M.iliocostalis	0.382	− 0.302	0.848
AUC_tHb_M.longissimus	0.532	0.020	0.834
AUC_tHb_M.multifidus	0.500	− 0.204	0.851
AUC_tHb_M.iliocostalis	0.176	− 0.772	0.800
**Median1_SmO**_**2**_**_M.longissimus**	**0.964**	**0.873**	**0.990**
**Median1_SmO**_**2**_**_M.multifidus**	**0.911**	**0.684**	**0.977**
Median1_**SmO**_**2**_**_**M.iliocostalis	0.003	− 0.631	0.694
Median1_tHb_M.longissimus	0.720	0.302	0.909
Median1_tHb_M.multifidus	0.553	− 0.118	0.869
Median1_tHb_M.iliocostalis	0.195	− 0.632	0.796
**Median2_SmO**_**2**_**_M.longissimus**	**0.986**	**0.941**	**0.997**
**Median2_SmO**_**2**_**_M.multifidus**	**0.905**	**0.640**	**0.978**
Median2_SmO_2__M.iliocostalis	0.253	− 0.508	0.838
Median2_tHb_M.longissimus	0.511	− 0.182	0.864
Median2_tHb_M.multifidus	0.556	− 0.180	0.882
Median2_tHb_M.iliocostalis	0.111	− 0.965	0.819
**Median3_SmO**_**2**_**_M.longissimus**	**0.983**	**0.933**	**0.996**
**Median3_SmO**_**2**_**_M.multifidus**	**0.908**	**0.653**	**0.979**
Median3_SmO_2__M.iliocostalis	0.500	− 0.389	0.894
Median3_tHb_M.longissimus	0.599	− 0.043	0.885
Median3_tHb_M.multifidus	0.716	− 0.032	0.946
Median3_tHb_M.iliocostalis	0.341	− 0.371	0.801
Recovery time_SmO_2__M.longissimus	0.252	− 0.620	0.779
Recovery time_SmO_2__M.multifidii	− 0.225	− 11.511	0.998
Recovery time_ SmO_2__M.iliocostalis	Incalculable		
Recovery time_tHb_M.longissimus	Incalculable		
Recovery time_tHb_M.multidifii	Incalculable		
Recovery time_tHb_M.liocostalis	Incalculable		

Recovery time for SmO_2_ *M. iliocostalis*, tHb *M. longissimus*, tHb *M. multifidus* and tHb *M. iliocostalis* could not be calculated due to a high number of missing data. Displayed are the intraclass correlation coefficients (ICC), the 95% confidence intervals (95% CI), lower level (LL) and upper level (UL).

Excellent reliability is highlighted in bold. Based on these results, only the measures for muscle oxygenation (SmO_2_) in the *M. longissimus* and *M. multifidus* were analysed in the main study.

### Main study

#### Demographic and clinical characteristics

142 participants were recruited for the main study. 26 of the CNSLBP patients were excluded because they were not able to perform the BSME test (e.g. due to high blood pressure) or because the subcutaneous tissue thickness exceeded 13 mm (n = 3). The asymptomatic subjects matched to these patients were also excluded to keep the matching pattern. Thus, the final study sample consisted of 90 participants; 45 CNSLBP patients and their individually-matched 45 asymptomatic controls. The groups demonstrated no differences in demographic and clinical characteristics, except for the BSME holding time, pain intensity and subcutaneous tissue thickness (Table 2). 17 of the asymptomatic subjects were able to maintain the BSME position for the maximum of 240 s, whereas only 7 of the CNSLBP patients completed the maximum.

**Table 2:** Demographic and clinical characteristics of the final sample including group comparison of the chronic non-specific low back pain (CNSLBP) group and asymptomatic controls.

Variable	CNSLBP patients (n = 45)	Asymptomatic controls (n = 45)	Group comparison LBP patients and asymptomatic controls (*P* and 95% CI (LL, UL))
Sex (male/female)	14/31	14/31	
Age (years) (mean, (SD))	34.02 (12.36)	33.16 (12.41)	0.744 (− 6.17, 4.44)
Height (cm) (mean, (SD))	170.44 (10.20)	170.24. (7.76)	0.910 (− 3.90, 3.48)
Weight (kg) (mean, (SD))	67.47 (11.68)	65.19 (9.84)	0.288 (− 6.55, 1.99)
BMI (mean, (SD))	23.16 (3.13)	22.40 (2.29)	0.197 (− 1.91, 0.40)
Subcutaneous tissue thickness (mm) (mean, (SD))	8.98 (2.76)	7.58 (2.39)	**0.012 (0.31, 2.48)**
Pain intensity (mean, (SD))	2.78 (1.79)	0 (0)	** < 0.0005 (**− **3.32,** − **2.24)**
Sport score (median (Q1,Q3))	3 (2,4)	3 (2.4)	0.965 (0.961, − 0.968)
BSME holding time (seconds) (mean, (SD))	164.64 (50.61)	189.60 (46.65)	**0.032 (2.31, 47.59)**

Displayed are significance (*P*), 95% confidence intervals (95% CI), lower limit (LL) and upper limit (UL).

Significant values are in bold.

#### Group comparison

**Figure 2:**
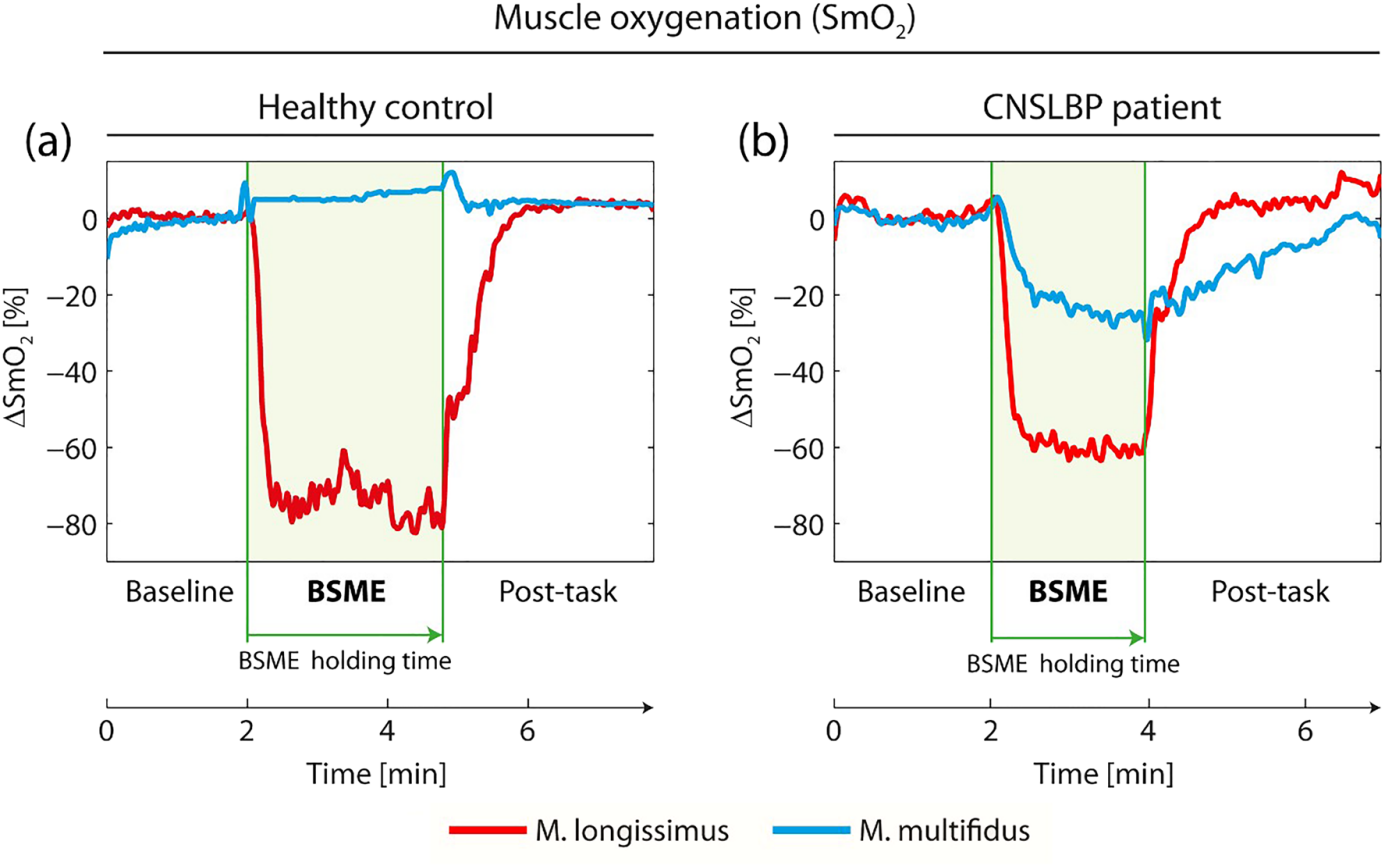
Muscle oxygenation of *M. longissimus* and *M. multifidus* of a representative asymptomatic subject (**a**) and a representative CNSLBP patient (**b**) before (0–2 min), during (2 to maximal 6 min) and after execution of the Biering–Sorensen muscle endurance (BSME) test. The green lines indicate the start and the end of the BSME test.

Qualitatively, the following time course of muscle oxygenation was observed for the *M. longissimus* in asymptomatic controls and CNSLBP patients: during the resting baseline period muscle oxygenation was stable. At the start of the BSME test, there was a rapid decrease of oxygenation reaching a plateau with only small variations in oxygenation. After cessation of the BSME test, muscle oxygenation increased rapidly and baseline levels were reached approximately 2 min after the end of the BSME test (Fig. 2a,b). Oxygenation of the *M. multifidus* in many asymptomatic subjects changed only very little during baseline, BSME test and recovery (Fig. 2a). Oxygenation in the *M. multifidus* in many CNSLBP patients seemed to decrease somewhat during the BSME test and to slowly increase after cessation of the test (Fig. 2b).

There was no statistically significant difference between patients and controls in oxygenation of the *M. longissimus* or *M. multifidus*, irrespective of whether age, gender and BMI were controlled for in the analysis (ANCOVA) or not (unpaired t-test, Table 3).

**Table 3:** Results of the unpaired t-tests of the unstandardized residuals (corrected SmO_2_) for area under the curve (AUC) and medians of NIRS measurements of *M. longissimus* and *M. multifidus* in chronic nonspecific low back pain subjects (CNSLBP) and asymptomatic controls.

Variables	Mean CNSLBP patients (SD)	Mean asymptomatic controls (SD)	Significance (*P*) and 95% CI (LL, UL)
AUC (SmO_2_) *M. longissimus* (∆SmO_2_ (%) × time (s))	− 193.71 (4148.23)	− 276.50 (4361.66)	0.936 (− 1963.68, 2129.26)
AUC (SmO_2_) *M. multifidus* (∆SmO_2_ (%) × time (s))	− 272.18 (3086.92)	− 77.55 (2633.75)	0.792 (− 1664.68, 1275.43)
Median 1 (SmO_2_) *M. longissimus* (%)	− 2.90 (23.13)	0.00 (21.98)	0.591 (− 13.67, 7.85)
Median 1 (SmO_2_) *M. multifidus* (%)	− 2.43 (17.45)	0.43 (18.17)	0.531 (− 11.97, 6.23)
Median 2 (SmO_2_) *M. longissimus* (%)	− 2.08 (23.54)	− 0.99 (22.79)	0.852 (− 12.67, 10.50)
Median 2 (SmO_2_) *M. multifidi* (%)	− 2.05 (17.69)	0.29 (15.10)	0.591 (− 11.07, 6.36)
Median 3 (SmO_2_) *M. longissimus* (%)	− 1.74 (24.89)	− 1.75 (23.39)	0.998 (− 10.95, 10.99)
Median 3 (SmO_2_) *M. multifidi* (%)	− 0.73 (15.47)	− 0.78 (16.87)	0.990 (− 7.34, 7.44)

Displayed are the mean, significance and 95% confidence intervals, lower limit (LL) and upper limit (UL).

To reproduce the analysis of Kell and Bhambhani^13^, only patients with pain intensity above 2 and the matched asymptomatic controls were included in a second analysis. This resulted in a reduced patient sample, which did not differ from the full sample with respect to any of the demographic or clinical variables, except for pain intensity (*P* 0.01). The reduced sample consisted of 24 CNSLBP subjects (mean age 30.88 (SD 9.24), 17 females, 7 males) and 24 asymptomatic controls (age 29.92 (SD 9.63), 17 females, 7 males)). Again, there were no statistically significant group differences in any of the NIRS parameters of the *M. longissimus* or *M. multifidus* in the unpaired t-test (Table 4) or the ANCOVA.

**Table 4:** Results of the unpaired t-test of the unstandardized residuals (corrected SmO_2_) for area under the curve (AUC) and medians of oxygenation of the *M. longissimus* and *M. multifidus* in chronic nonspecific low back pain subjects (CNSLBP) with pain ratings above 2 and asymptomatic controls.

Variables	Mean CNSLBP patients (pain intensity above 2)	Mean asymptomatic controls	Significance (*P*) and 95% CI (LL, UL)
AUC (SmO_2_) *M. longissimus* (∆SmO_2_ (%) × time (s))	715.32 (3097.27)	− 1101.59 (4840.35)	0.194 (− 976.33, 4610.17)
AUC (SmO_2_) *M. multifidus* (∆SmO_2_ (%) × time (s))	− 458.23 (2967.43)	− 914.42 (3157.92)	0.678 (− 1764.52, 2676.91)
Median 1 (SmO_2_) *M. longissimus* (%)	1.11 (17.21)	− 1.47 (25.07)	0.723 (− 12.51, 17.33)
Median 1 (SmO_2_) *M. multifidus* (%)	− 4.35 (17.86)	− 3.71 (20.49)	0.926 (− 14.50, 13.22)
Median 2 (SmO_2_) *M. longissimus* (%)	1.14 (19.33)	− 4.15 (24.52)	0.513 (− 11.02, 21.61)
Median 2 (SmO_2_) *M. multifidi* (%)	− 6.01 (18.99)	− 4.14 (16.55)	0.783 (− 15.71, 11.97)
Median 3 (SmO_2_) *M. longissimus* (%)	2.95 (17.91)	− 3.87 (25.92)	0.344 (− 7.61, 21.27)
Median 3 (SmO_2_) M. multifidi (%)	− 4.14 (15.57)	− 1.09 (17.67)	0.575 (− 14.01, 7.90)

Displayed are the mean, significance (*P*) and 95% confidence intervals, lower limit (LL) and upper limit (UL).

#### Multiple regression

The influence of muscle oxygenation of the *M. longissimus* and the M. multifidi (corrected SmO_2_) on BSME holding times was tested, in addition to the influence of sex, physical activity, BMI, group (LBP/asymptomatic controls), MAP, PP, HR and pain intensity. Because the residuals of the medians were highly correlated (Pearson correlation coefficients ≥ 0.7), separate models were calculated for each median. The model for median 1 showed a statistical trend (F(11,38) = 1.89, P = 0.072). The model for median 3 was statistically significant, the model for median 2 was neither statistically significant and did not show a trend. The amount of variance explained was around 37%, with significant predictions from the sport score and BMI (shown for median 3 in Table 5). In none the models did the muscle oxygenation parameters or systemic physiological measures significantly influence BSME holding times.

**Table 5:** Multiple regression results for BSME shown for Median 3.

BSME	B	95% CI for B	SE B	β	R^2^	ΔR^2^	Sig
Model					0.372	0.248	0.003
Constant	243.643	139.80 to 347.482	51.835				
Pain intensity	− 6.090	− 14.723 to 2.542	4.309	− 0.245			0.163
Group	− 1.205	− 33.305 to 30.894	16.024	− 0.013			0.940
Age	0.784	− 0.210 to 1.778	0.496	0.199			0.120
BMI	− 7.028	− 11.127 to − 2.929	2.046	− 0.412			**0.001**
Sex	1.928	− 22.192 to 26.047	12.040	0.020			0.873
Sport score	14.759	2.157 to 27.360	6.291	0.264			**0.023**
Residual Median 3 *M. longissimus* (SmO_2_)	0.174	− 0.264 to 0.611	0.218	0.089			0.430
Residual Median 3 *M. multifidus* (SmO_2_)	0.052	− 0.618 to 0.721	0.334	0.018			0.878
Pulse pressure	0.034	− 1.225 to 1.293	0.628	0.012			0.957
Mean arterial pressure	0.230	− 1.968 to 2.427	1.097	0.052			0.835
Heart rate	0.457	− 0.279 to 1.193	0.367	0.195			0.219

Displayed are B = unstandardized regression coefficient, CI = confidence interval, SE B = standard error of the coefficient, β = standardized coefficient, R^2^ = coefficient of determination, ΔR^2^ = adjusted R^2^ and Sig. = significance.

Significant values are in bold.

#### Power considerations

None of the SmO_2_ measures showed any significant difference between the groups in the present study. Previous studies examining differences in NIRS parameters between LBP patients and controls had relatively small sample sizes [29 (12 healthy/17 CLBP patients) and 68 (34 healthy/34 CLBP patients)]^3,6^. The sample of the current study was based on these previous studies with the goal of generously exceeding it. With a sample size of 90 (45 per group), a significance level of alpha = 0.5, and an observed effect size of 0.33, a power (1-beta) of 0.8 was reached (G*Power, Dusseldorf, Germany^36^).

## Discussion

The first aim of this study was to compare BSME holding times as well as muscle perfusion and oxygenation during the BSME test between CNSLBP patients and asymptomatic controls individually matched for age, BMI, *and* physical activity. The second aim was to investigate the influence of muscle oxygenation, systemic physiological measures and subcutaneous tissue thickness on BSME holding times. Beforehand, reliability of the NIRS measurements was assessed in a separate sample to use the most reliable measures for analysis of the main study. The most reliable measures, achieving ‘excellent reliability’^34^, were muscle oxygenation of the *M. longissimus* and *M. multifidi*. Measures of the iliocostalis muscle were excluded from further analysis because of poor reliability and a considerable amount of missing data, in some instances due to space restrictions on the subjects’ back or because the data acquisition of the probe was arguable due to flatlined read-out during measurement. Muscle perfusion showed poor to moderate reliability for all three muscles and was therefore also excluded from further analyses^34^.Thus, only the muscle oxygenation data of *M. longissimus* and *M. multifidi* were used for further statistical analysis. Despite a statistically significant difference in BSME holding times between CNSLBP patients and asymptomatic controls, there was no difference in muscle oxygenation between the groups. This finding was confirmed in a sub-sample of CNSLBP patients with a pain intensity above 2. NIRS measurements were influenced by subcutaneous tissue thickness, which was addressed by correcting SmO_2_ for subcutaneous tissue thickness before using it in further analyses. Multiple regression analysis revealed that BSME holding times were influenced by BMI and sport scores, but not by muscle oxygenation.

### Reliability

Kell et al. observed excellent reliability during the BSME for back muscle oxygenation and perfusion (ICC 0.96 and 0.95)^37^. The present study supports these findings by showing excellent reliability of muscle oxygenation in *M. longissimus* and *M. multifidus*. In contrast, the reliability of muscle perfusion was poor to moderate. Several differences between the previous reliability study and the present one might contribute to this discrepancy. Body and muscle composition, which itself is influenced by age and sex, is known to influence NIRS measurements^30,38,39^. Kell et al. only measured males, whereas the present sample was mixed. In addition, the sample in the present study was on average 7.6 years older. The technical set-up also differed slightly: in Kell et al., the probes were placed bilaterally at the height of L3 without specifying the underlying muscles. In contrast, the present study adhered to the SENIAM guidelines that were developed for the placement of surface electromyography^22^ and measured *M. longissimus*, *M. multifidus* and *M. iliocostalis* separately within an area from L5 to L1. Given that the oxygenation of the *M. longissimus* and the *M. multifidus* behaved differently, this approach seems justified. The poor reliability of the *M. iliocostalis* is in line with other surface measurement modalities^40,41^. These findings might generally be related to reduced reliability of palpation of *M. iliocostalis* because of e.g. variation of orientation^42^ and possibly missing the muscle when placing the NIRS probe. Furthermore, the present study used different parameters for analysis, i.e. AUC and medians. These measures are more robust with respect to outliers compared to using simply the minima and maxima as in Ref.^43^. Taken together, it seems important that reliability measurements are conducted in a sample of similar age and sex distribution and with an identical technical set-up as in the research study itself, as was the case here.

### Main study

The findings of a shorter BSME holding time in CNSLBP patients is in line with current literature as the BSME is proposed to discriminate well between LBP subjects and asymptomatic controls^10^. However, none of the NIRS oximetry parameters that were found to be reliable differed between patients and controls. This was true irrespective of whether age, gender and BMI were corrected for or not. In accordance with this finding, none of the muscle oxygenation parameters explained a significant amount of variance of BSME holding times. These findings are identical to a previous study by McKeon et al.^11^, although in that study no matching for physical activity between patients and controls was performed. However, the results of the present study and the study by McKeon et al. conflict with the study by Kell and Bhambhani that investigated the BSME and back muscle oxygenation/perfusion in CNLBP patients^13^. In that study, a difference in recovery time was observed between healthy subjects and two groups of CNSLBP patients (active and sedentary)^13^. Surprisingly, BSME holding times did not differ in the study by Kell and Bhambhani, in contrast to a large body of literature, including one review^12,14,16,25^. A difference between the study by Kell and Bhambhani and the present one was that patients had to have pain greater than 2/10. Because this was not an inclusion criterion in the present study, the analysis was repeated using a sub-sample of patients with pain greater than 2/10. Similar to the full sample, the reduced patient sample had shorter BSME holding times and showed no difference in the NIRS oximetry parameters compared to the controls, thereby still not replicating the previous findings. It has to be noted, however, that recovery time was not analyzed in the present study, because its reliability was not found to be high enough (reliability was not assessed in the study by Kell and Bhambhani). This reiterates the importance of incorporating reliability measurements of the exact experimental set-up in NIRS muscle oximetry studies.

So how can reduced BSME holding times in NSCLBP patients be explained, when back muscle oxygenation does not differ from that of controls? Because physical activity levels are known to influence the holding capacity of the lumbar extensor muscles^18^, but are typically not assessed and/or not considered in the analysis of NIRS muscle oximetry studies of CLBP patients^3–6,11,13^, this study individually matched also for activity levels. We found a relationship between activity levels and BSME holding times in the regression analysis, but NIRS oximetry parameters did not differ between patients and controls. Additionally, we used systemic physiological measures in our regression models, but were not correlated with BSME holding times. Only a small percentage of variance was explained by the variables used in the regression analysis.

This points to two conclusions: First, BSME holding times are reduced in NSCLBP patients because of some other factor that was not measured in the present study, such as reduced motivation^17,44^ or different recruitment of muscles beyond back extensors^10,45^. Second, it appears unlikely that altered back muscle oxygenation and subsequent accumulation of metabolites is a major source of pain in patients with CNSLBP.

Two more aspects have to be kept in mind, when working with NIRS muscle oximetry measurements in general. First, measurements are influenced by the subcutaneous tissue thickness of the test subjects^23,46,47^. The present study adhered to the recommendation of the manufacturer, and only included test subjects with a subcutaneous tissue thickness of less than 13 mm. Nevertheless, there was a significant influence of tissue thickness on NIRS measures. It can therefore be concluded that it is not sufficient to adhere to the manufacturer’s guidelines. Interestingly, although patients and controls were matched for BMI, subcutaneous tissue thickness was greater in patients than in controls and it was therefore important to control the NIRS measures statistically for tissue thickness. Second, NIRS oximetry measurements also depend on the measurement device^21^. Both studies which preceded the present study used different NIRS devices^11,13^, possibly contributing to the divergent findings.

## Limitations

In this study physical activity was assessed using a self-reported questionnaire, which might not reflect the real physical activity status of participants^48^. Additionally, the age of the participants was relatively young. Because muscle perfusion changes with age^49^, it is conceivable that differences in back muscle perfusion might only become apparent in older patients with CNSLBP. Furthermore, the SENIAM guidelines were developed for the use of electrode placements of surface electromyography and have not been validated for the use of NIRS probes. Lastly, because of the poor reliability of the NIRS measures of the *M. iliocostalis*, we cannot comment on any potential group differences of NIRS measures in this muscle.

## Conclusion

NIRS oximetry measures of the *M. longissimus* and *M. multifidus* were found to be reliable measures during the BSME test. When comparing patients with CNSLBP to an individually-matched control sample, no difference in the oxygenation of back muscles during the BSME was detected. Thus, at this point, insufficient oxygenation during back muscle endurance does not seem to be a major pain mechanism in relatively young patients with CNSLBP. Furthermore, muscle oxygenation and systemic physiological measures did not influence BSME holding times. Therefore, future investigation should ensure to evaluate motivational determinants of BSME holding times as well as the recruitment of auxiliary muscles.

## Data Availability

The datasets generated during and/or analysed during the current study are available from the corresponding author on reasonable request.
